# Context-Dependent Mutation Dynamics, Not Selection, Explains the Codon Usage Bias of Most Angiosperm Chloroplast Genes

**DOI:** 10.1007/s00239-021-10038-w

**Published:** 2021-12-21

**Authors:** Brian R. Morton

**Affiliations:** Department of Biology, Barnard College, Columbia University, 3009 Broadway, New York, NY 10027 USA

## Abstract

Two competing proposals about the degree to which selection affects codon usage of angiosperm chloroplast genes are examined. The first, based on observations that codon usage does not match expectations under the naïve assumption that base composition will be identical at all neutral sites, is that selection plays a significant role. The second is that codon usage is determined almost solely by mutation bias and drift, with selection influencing only one or two highly expressed genes, in particular *psbA*. First it is shown that, as a result of an influence of neighboring base composition on mutation dynamics, compositional biases are expected to be widely divergent at different sites in the absence of selection. The observed mutation properties are then used to predict expected neutral codon usage biases and to show that observed deviations from the naïve expectations are in fact expected given the context-dependent mutational dynamics. It is also shown that there is a match between the observed and expected codon usage when context effects are taken into consideration, with *psbA* being a notable exception. Overall, the data support the model that selection is not a widespread factor affecting the codon usage of angiosperm chloroplast genes and highlight the need to have an accurate model of mutational dynamics.

**Supplementary Information:**

The online version contains supplementary material available at 10.1007/s00239-021-10038-w.

## Introduction

Codon usage bias, or CUB, arises from an interplay of mutation bias plus drift, and selection (Morton 2003; Sharp et al. 2005, 2010; Hershberg and Petrov 2008), and a common issue in genomics is to determine the degree to which selection affects CUB of genes within a given genome. One approach that is frequently used to draw inferences about the impact of selection is to compare the pattern of base composition at synonymous sites, essentially the pattern of CUB, to an expectation that is assumed to represent neutrality. For example, a standard measure of CUB is the Effective Number of Codons (ENC) statistic (Wright 1990) that represents the degree of deviation from uniform codon usage across all degenerate groups. It is common to compare the ENC value of a gene to the ‘expected’ ENC that would arise from G + C composition bias. This can be calculated using an estimate of neutral G + C content of synonymous sites, such as the G + C content of third codon position sites (GC3), G + C content of fourfold degenerate sites (GC3S), genome G + C or intergenic G + C (Wright 1990). The relationship between G + C and ENC, referred to herein as ENC_E_, is essentially a standard curve and the comparison of genes to this curve is commonplace, with deviation from the curve taken as evidence for an influence of selection on codon usage (e.g., Yang et al. 2014; Wei et al. 2014; Wu et al. 2015; He et al. 2016; Hussain and Rasool 2017; Wang et al. 2018; Guan et al. 2018; Muthabathula et al. 2018; Li et al. 2019). Another compositional feature that is commonly used as an estimate of neutrality is Parity Rule 2, or PR2 (Sueoka 1995). In this case the expectation, what will be called PR_E_, is that N_A_ = N_T_ and N_G_ = N_C_ at neutral fourfold degenerate sites along the coding strand of a gene, with significant deviation from PR_E_ taken as evidence for selection (e.g., Duan et al. 2021).

Both ENC_E_ and PR_E_ have been utilized in several studies of CUB in angiosperm chloroplast DNA (cpDNA), and the observation that there is widespread deviation between ‘expected’ and observed CUB has been used to argue that selection plays a significant role in shaping the CUB of chloroplast genes (Zhang et al. 2007, 2018; Xu et al. 2011; GuangXin et al. 2020; Liu et al. 2020; Duan et al. 2021). This assertion about selection conflicts with earlier analyses of angiosperm chloroplast genomes, which developed a model of CUB with a limited role for selection (Morton 1993, 1998, 2003; Suzuki and Morton 2016). In this second model, CUB of angiosperm chloroplast genes is determined almost solely by mutation bias plus drift, with the exception of the highly translated *psbA* gene, which codes for the core protein of photosystem II, a protein that turns over at an extremely high rate and is the major translation product (Mullet and Klein 1987) and the main protein-coding transcript (Castandet et al. 2016) in the chloroplast, and possibly the highly expressed *rbcL* gene which codes for the large subunit of RuBisCO (Morton 1998; Suzuki and Morton 2016).

The basis of this second model is that the CUB of angiosperm *psbA* genes is atypical relative to other angiosperm chloroplast genes, most noticeably in that it has a bias towards C at the third position of twofold degenerate NNY codon groups (Phe: TTY, Tyr: TAY, Cys: TGY, His: CAY, Asn: AAY, and Asp:GAY), a bias that runs counter to the genome A + T bias. The CUB pattern of *psbA* matches the limited tRNA population of the chloroplast genome, and also matches the CUB pattern of several highly expressed genes in green algae such as *Chlamydomonas reinhardtii* (Morton 1998). For example, although the *C. reinhardtii* genome is roughly 35% G + C overall (Maul et al. 2002) and fourfold degenerate sites are strongly biased towards A + T, 93.8% of the codons for the twofold degenerate amino acids Phe, Tyr, Cys, His, Asn, and Asp in the *C. reinhardtii psbA* gene have a C at the degenerate third position, and fourfold degenerate codon groups are strongly biased towards the codon with a T at the third position (Morton 1998, 2003).

The atypical CUB of the angiosperm *psbA* gene can be quantified using the Codon Adaptation Index (CAI from Sharp and Li 1987) to measure CUB. This directional statistic requires an estimate of codon fitness values, typically taken from genes thought to be under strong selection, and measures the degree to which a given gene utilizes these high fitness codons. When highly expressed green algae genes, such as *psbA* and *rbcL* from *C. reinhardtii*, are used to estimate fitness values, the angiosperm *psbA* gene has a higher CAI value than other angiosperm chloroplast genes although it is much lower than green algae chloroplast genes (Morton 1998). Altogether, these observations have led to the proposal that the *psbA* pattern of CUB is the result of codon adaptation, or selection for translation efficiency, and that codon adaptation results in a bias towards the same adaptive CUB in all plastid lineages, with selection being much weaker in angiosperms than in algae, essentially limited to *psbA* (Suzuki and Morton 2016).

Although it is possible that these two models are not mutually exclusive, it is not likely that both can be true. If selection for translation efficiency results in the CUB observed in highly expressed green algal chloroplast genes, and only *psbA* shows a bias towards this pattern, then what widespread selective pressure could be influencing the codon usage of other angiosperm chloroplast genes? On the other hand, if selection for translation efficiency is widespread in most angiosperm chloroplast genomes, then what force is driving the atypical CUB of *psbA*?

Here, the use of ENC_E_ and PR_E_ in the study of selection on CUB is examined and it is shown that these approaches can be misleading since they rely on the assumption that the mutation process will evolve identical base composition across all neutral sites. However, mutation dynamics, that is the relative probabilities of different mutations, can be context-dependent, meaning that they vary as a function of the base composition of flanking nucleotides. Context dependency has been observed in many genomes (Morton 1995, 2003; Arndt et al. 2003; Arndt and Hwa 2005; Sung et al. 2015; Zhu et al. 2017; Aikens et al. 2019; Ling et al. 2020) and could lead to significant deviations from the assumption that all neutral sites will evolve to the same base composition. Although the angiosperm chloroplast genome is used as an example here, the results are more generally applicable and make it clear that a consideration of complex mutation models is necessary if composition features are used to analyze the role of selection at synonymous sites.

The approach used here is based on an earlier study (Morton 2003) that examined codon usage in the light of context-dependent mutation dynamics. The basic findings of the previous study are expanded to explicitly examine how context dependency can affect the use of certain measures of codon usage and base composition to make inferences about selection. It has long been known that there is significant heterogeneity in substitution dynamics across intergenic sites of the angiosperm chloroplast genome as a function of local context (the base composition of flanking nucleotides). Both A + T content of flanking bases and the distribution of purines across the strands at neighboring sites have a profound impact on the relative rates of different substitutions (Morton and Clegg 1993, 1995; Morton 1995, 2003). These dynamics are observed in both intergenic regions and at fourfold degenerate sites of genes suggesting that they are a feature of the underlying mutation dynamics (Morton 2003). The result of this type of heterogeneity is that there is no single expected composition bias. Instead, the expected base composition at equilibrium varies widely across neutral sites and is in flux as neighboring bases mutate.

The current study examines how context dependency of mutations affects analyses of selection on codon usage and selection more generally. Data from intergenic regions of closely related angiosperm chloroplast sequences are used to derive context-dependent substitution matrices as a function of the tetranucleotide context, that is the four bases, two on each side, surrounding the site of substitution. The stationary vector for each matrix, which is equivalent to the equilibrium base composition, is then derived and it is observed that dramatically different base compositions would evolve by genetic drift across neutral sites in different contexts. These substitution matrices are then used to derive expected CUB and composition patterns, that is the patterns that would evolve in the absence of selection, of each angiosperm chloroplast gene given the amino acid sequence, which determines the tetranucleotide context of each third codon position. It is shown that the context-dependent substitution patterns lead us to predict strong and consistent deviation from both ENC_E_ and PR_E_ in the absence of selection, indicating that these measures are not accurate estimates of neutrality. A nested resampling of substitutions from matrices is then employed to predict expected distributions of codon usage and base composition statistics of individual genes in the absence of selection. In essence, we test the null hypothesis that mutation bias and drift can explain CUB of chloroplast genes. For every gene except *psbA* we do not reject the null. Both the observed CUB and the observed base composition features at synonymous sites fall within the expected ranges predicted by the context-dependent mutation dynamics. There is also a slight but significant deviation in CAI for *rbcL* and *psbD* suggesting they may also be under weak selection.

The results strongly support the second model of CUB in angiosperm chloroplast genes. When the complexity of mutations in cpDNA is accounted for there is no evidence that selection has a widespread influence on the CUB of angiosperm chloroplast genes. Instead, fixation by random genetic drift of selectively neutral, but context-dependent, mutations can account for the observed patterns of codon usage in almost all genes. The results also indicate that caution needs to be employed when using composition data to predict patterns such as CUB in the absence of selection. Uniformity in equilibrium composition across neutral sites cannot be assumed but, rather, must be demonstrated by showing that context does not impact mutation dynamics, before any inferences are drawn about selection. In the case of the angiosperm chloroplast genome this assumption is not valid.

## Methods

### Mutation Models

RefSeq complete angiosperm chloroplast genome sequences were downloaded from NCBI (www.ncbi.nlm.nih.gov/genome/browse#!/eukaryotes/) on March 14, 2019 and then parsed with the Biopython 1.76 (Cock et al. 2009). Regions between neighboring Biopython SeqRecords were saved as intergenic regions. Genomes were grouped into closely related triplets based on the taxon information in the NCBI files by selecting two genomes at random from each genus that had at least two representative genomes and then selecting an outgroup taxon at random from outside that genus but within the same Family. This yielded a total of 280 sequence triplets from 39 different families.

Intergenic regions were aligned using the Clustal alignment function in Biopython with a gap open penalty of 2 and a gap extend penalty of 0.5. Regions with an alignment greater than 70 nucleotides in length were retained. Within intergenic regions only sites that met the following criteria were used in the analysis: within the 10 bases surrounding the site, five on each side, there had to be at least 8 base pairs in the ingroup sequences (i.e., no gaps in either sequence) and the two ingroup sequences needed to show at least 70% similarity. These were implemented to reduce comparisons of nonhomologous sequences that are within the aligned regions and should not bias inference of mutation parameters unless the context effects vary significantly between conserved and more variable regions (Morton 1995). Protein-coding sequences were aligned with a gap open penalty of 5 and a gap extend penalty of 2.

From these sequences, with the restrictions just described, a substitution matrix was generated for each possible tetranucleotide context, defined as the base composition of the two immediate 5′ and two immediate 3′ neighbors of the substitution. All sites in an intergenic alignment and all fourfold degenerate sites in protein-coding regions with a conserved tetranucleotide context in all 3 sequences were scored. Sites that were conserved between the ingroup pair were scored as a conserved site (e.g., A → A) while those that differed in the ingroup sequences were scored as an N_a_ → N_d_ substitution, where N_a_ is the ancestral nucleotide, inferred from the outgroup, and N_d_ the derived. If all 3 sequences differed the site was ignored. The matrix rows represent N_a_ and the columns represent N_d_. This analysis was performed by a Python program generated by the author.

Once the 256 substitution matrices had been generated, complementary matrices were combined. For example, the AG_GA context and the TC_CT context matrices (where _ indicates the substitution site and the bases indicate the flanking nucleotides 5′ and 3′) were combined by adding the complementary matrix of one to the other. The stationary vector Φ of each matrix Π, representing the equilibrium base frequency for a sequence evolving with the mutation dynamics given by the matrix, was calculated by generating Π^t^ for large t, each row of which will equal Φ (Cox and Miller 2017), such that Φ=ΦΠ. A pseudocount entry of one substitution was inserted in the case of no observed substitutions of a given type in any context for this calculation. All calculations were done using Python script written by the author.

### Matrix Comparisons

Substitution dynamics within different contexts were compared using a resampling test. This process generated a set of pairs of resampled matrices based on the null hypothesis that they are drawn from the same underlying set of substitution probabilities. A distance value for the original pair is then compared to the distribution of distance values of the resampled pairs.

For the two matrices being compared, a rate matrix was calculated for each by dividing each value by the relevant row sum. The distance between the two matrices was calculated as the sum of the squared differences between the off-diagonal (substitution) rate values. The two matrices were then concatenated and the frequency of each mutation within calculated relative to the sum of off-diagonals in the same row. Two new matrices were then generated by resampling. In each case, the resampled matrix had the same number of off-diagonal entries within each row as did the original matrix. The distance between the resampled pairs was then calculated as described for the original pair. This resampling was repeated 100 times and the position of the original matrix distance within the distribution of resampled matrix pair distances was determined. If the distance between the two matrices lies within the top 5% of the resampled distribution then the null hypothesis, that the substitutions in the two contexts are drawn from the same set of substitutions as estimated by the combined matrices, is rejected. No multiple test correction was performed since the goal was not to determine which pairs were significant but just to determine if the frequency of rejection was above the expected 5% level.

### Expected Base Composition and Codon Usage

For each protein-coding sequence from the *Zea mays* genome greater than 300 codons in length, extracted as described above, the Effective Number of Codons (ENC) was calculated (Wright 1990). A gene that used just one codon per synonymous group, the strongest possible bias, would have an ENC of 20, while a gene with an equal number of codons within each synonymous group, no CUB, would have an ENC of 61. ENC does not represent direction of bias; that is, it does not assess which codons are utilized more frequently only the degree of deviation from uniform codon usage. PR-AT and PR-GC [N_A_/(N_A_ + N_T_) and N_G_/(N_G_ + N_C_), respectively] for fourfold degenerate sites, as well as C_2_, the NNC content of twofold degenerate codon groups (i.e., TTC, TAC, TGC, CAC, AAC, and GAC) were also calculated.

Expected codon usage of each gene was calculated for each gene as a function of the equilibrium base frequencies calculated above. For each codon, the tetranucleotide context of the third position was determined. This is the first two positions of that codon and the first two positions of the downstream codon. For degenerate codons, the stationary vector of the matrix from that context was then taken as the expected frequency of each nucleotide at that site, which implicitly assumes the site is at equilibrium. In the case of twofold and threefold degenerate sites, the expected frequency of each base was taken as the fraction of that base given the synonymous bases that could occur at that site. From this expected codon usage, what are referred to here as the context-dependent expectations ENC_CD_ and PR_CD_ were calculated. Also calculated was CAI_CD_, the context-dependent Codon Adaptation Index (Sharp and Li 1987) of this expected codon usage, using cumulative codon frequencies from the *Chlamydomonas reinhardtii psbA* and *rbcL* genes as fitness values. The use of the *C. reinhardtii* genes is based on the observation that these highly expressed green algal genes show strong evidence for codon adaptation and that the codon bias of these genes is optimal for translation efficiency across all plastids (Morton 1998; Suzuki and Morton 2016). Therefore, the CAI value calculated will measure the degree to which a gene utilizes codons that are optimal for translation in the chloroplast (Suzuki and Morton 2016). All calculations, including codon counts of gene sequences, were done using Python script written by the author.

### Resampling Tests of Observed Codon Usage

To test for significant deviation of a gene sequence from expected codon usage and base composition features, a nested matrix and codon resampling protocol was used consisting of 100 matrix resampling iterations within which there were 100 codon resampling iterations. For this analysis, 48 substitution matrices, generated by combining sets of the original 256 matrices in order to increase sample size, were used. Two combinations were performed and studied separately. In each case, all matrices with the same two immediate flanking bases and the same general composition features at the two nucleotides on site removed from the substitution were combined. In the first case, the general composition feature was A + T content, either 0, 1, or 2, in the second it was Y content, also either 0, 1, or 2. For example, in the first case, the AG_GA, TG_GA, TG_GT, and AG_GT matrices, all with G_G immediately flanking the site, were combined since they share an A + T = 2 context at the sites one nucleotide removed. Since the effect of the immediate neighbors on substitution heterogeneity is much stronger than sites one nucleotide away (data not shown), combining matrices provides a larger sample size while retaining the context effect.

In each of the 100 matrix resampling iterations, a set of 48 new context-dependent substitution matrices were generated by sampling with replacement from the 48 observed matrices. Each resampled matrix had the same number of changes in each row as the original matrix and the probability of sampling was the relative probability of that change in the original matrix. The set of 48 matrices was then used to generate 100 sequences with the same amino acid sequence as the gene. For each position, a synonymous (or identical for nondegenerate amino acids) codon was selected at random by determining the tetranucleotide context of the third position and finding the equilibrium vector the matrix of that context. Codons coding for a sixfold degenerate amino acid were treated as either the fourfold degenerate group or the twofold degenerate group depending on which existed in the gene sequence itself. The new codon was then selected with a probability determined by the relative equilibrium frequencies of those bases that would result in the same amino acid at that site as in the original sequence. This nested resampling generates a set of 10^4^ sequences within a space encompassing sampling error of our substitution matrices.

For each sequence, four statistics were calculated; PR-AT at fourfold degenerate sites, C_2_, Effective Number of Codons (ENC), and CAI as described above. For each statistic, an expected distribution is generated from the 10^4^ sequences, and this can be used for comparison to the observed value for that gene.

### Cluster Analysis

To cluster a set of genes by similarity in codon usage, a distance was calculated for each pair of genes using the following equation, where *f*^*x*^_ij_ is the frequency in gene *x* of codon *i* relative to the total number of codons (*n*) for amino acid *j* in the sequence, and *Na* is the number of degenerate amino acids (i.e., excluding Met and Trp) with at least one representation in each sequence.

$$d_{{12}} = \frac{{\sum\nolimits_{{j = 1}}^{{18}} {} \sum\nolimits_{{i = 1}}^{n} {(f_{{ij}}^{1} - f_{{ij}}^{2} )^{2} } }}{{N_{a} }}$$

The matrix of distances for all gene pairs was then clustered using the default hclust function within the R cluster package (Maechler et al. 2018).

## Results

### Heterogeneity of Mutation Dynamics and Local Context

A total of 4,938,691 sites and 37,595 substitutions (0.76% of sites) were scored in the comparisons of 280 ingroup pairs. The low rate of substitution indicates that the probability of multiple hits is low enough that our substitution matrices should be an accurate representation of the instantaneous transition matrices in noncoding regions and at fourfold degenerate sites in coding sequences. Previous studies of substitutions in angiosperm cpDNA have found that these two classes of fourfold degenerate sites show very similar general patterns of context dependency (Morton 2003) and a comparison of the matrices in this study shows highly correlated context features between coding and noncoding sequences. Additionally, all analyses presented below were repeated using matrices from just noncoding DNA with essentially the same results (data not shown). Therefore, these matrices are concluded to be a good approximation of the instantaneous mutation dynamics in angiosperm cpDNA and the analyses presented below used the combined matrices from coding and noncoding DNA to increase sample size.

As has been observed elsewhere regarding substitutions in angiosperm cpDNA, these mutations are strongly context-dependent. Previously it has been showed that both Ts:Tv and overall rate are strongly influenced by the A + T content of the two sites immediately flanking a substitution in angiosperm chloroplast sequences (Morton 1995; Morton et al. 1997). The current study develops a more detailed context-dependent mutation model since every possible tetranucleotide context was scored, along with an outgroup taxon to determine direction of change. An illustration of the variation across contexts can be seen in the comparison of the matrices for the CC_CC, CC_AA, and AA_AA contexts in Table 1. (Contexts are indicated by the two 5′ flanking bases, _ for the mutation site, followed by the two 3′ flanking bases.) One notable difference between them is in the transition:transversion (ts:tv) ratios. In the CC_CC context there is a strong bias towards transitions, much higher than in the other contexts. This is consistent with general relationship between flanking A + T and ts:tv that has been observed previously (Morton 1995, 1997).

**Table 1:** Three representative context-dependent mutation matrices

Context^a^				To	
CC_CC		A	C	G	T
	A	13,766	43	134	1
From	C	24	12,031	13	132
	G	155	27	6424	18
	T	3	140	23	12,530
	Equil	A = 26.4%	C = 32.7%	G = 11.3%	T = 29.6%
CC_AA		A	C	G	T
	A	16,566	62	39	27
From	C	76	6168	6	45
	G	68	17	5514	30
	T	22	33	31	13,269
	Equil	A = 43.4%	C = 13.5%	G = 9.4%	T = 33.7%
AA_AA		A	C	G	T
	A	63,064	38	56	77
From	C	48	8949	25	40
	G	124	21	20,755	228
	T	151	41	174	32,101
	Equil	A = 65.4%	C = 5.9%	G = 9.9%	T = 18.8%

The observed number of associations between the two in group sequences are given, as is the equilibrium base composition that would evolve given the mutation model of that matrix

^a^Context is given as N_5_N_5__N_3_N_3_ where _ is the site of observed substitution, N_5_N_5_ represents the two 5’ flanking bases and N_3_N_3_ the two 3’ flanking bases

More importantly here, the stationary vector in each of these three contexts is also shown in Table 1. The stationary vector is equivalent to the base composition at equilibrium that would evolve given the mutation scheme of that matrix. The differences between these equilibrium compositions show that the evolutionary trajectory of sites within the different contexts will be widely divergent. One effect of this, with respect to codon usage, is that there is no single expected codon usage for an amino acid or synonymous group. For example, in proline codons (CCN) upstream from another proline codon, the third codon position is in the CC_CC context and so these codons will evolve towards a very different codon usage than proline codons upstream from an asparagine (AAY) or a lysine (AAR) codon, where the third position is in the CC_AA context.

**Fig. 1:**
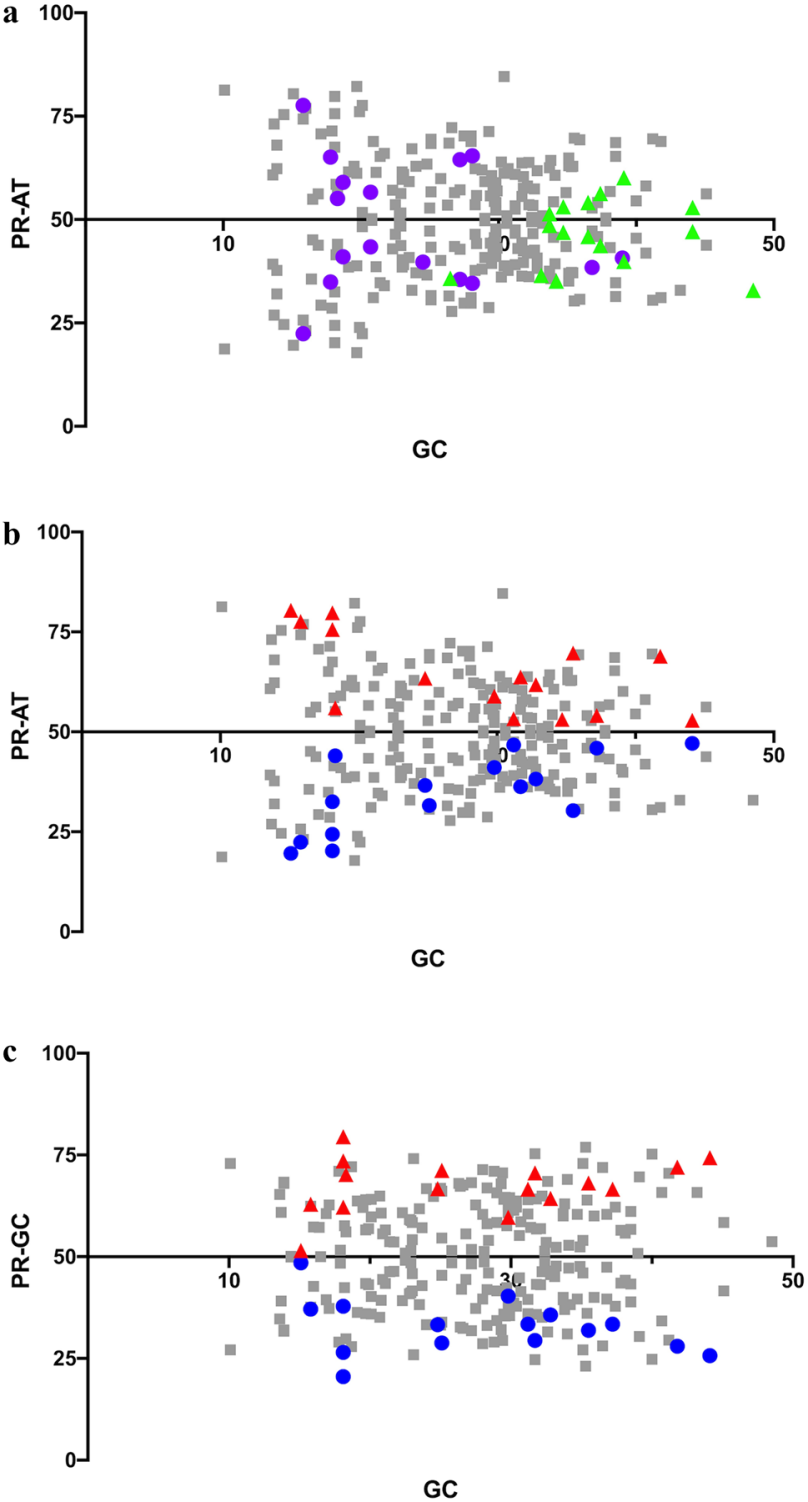
Base composition at equilibrium for sites within each of the 256 tetranucleotide contexts. **a** and **b** Both show %GC plotted against PR-AT [(N_A_)/(N_A_ + N)] with context features highlighted differently in the two in order to illustrate the relationship between context and mutation dynamics. In **a** contexts with 4 AT base pairs are plotted in purple, contexts with 4 GC base pairs are plotted in green. In **b** contexts with 4 pyrimidines on the analyzed strand are plotted in blue, contexts with 4 purines on the strand analyzed are plotted in red. **c** %GC plotted against PR-GC. Contexts with 4 pyrimidines on the analyzed strand are plotted in blue, contexts with 4 purines on the strand analyzed are plotted in red (Color figure online)

The full heterogeneity of mutation dynamics across all tetranucleotide contexts, represented by using features of the stationary vectors of the matrices, is illustrated in Fig. 1. Across contexts, the equilibrium composition ranges from 10.2% G + C to 48.5% G + C, while PR-AT [(N_A_)/(N_A_ + N_T_)] ranges from 17.8 to 84.6 and PR-GC ranges from 20.5 to 79.5. There are patterns to the relationship between context and mutation bias. For example, sites in higher A + T contexts tend to evolve to higher equilibrium A + T contents (Fig. 1A), and sites with more flanking pyrimidines on the same strand evolve to stronger pyrimidine skews on that strand (Figs. 1B and C). Overall, the data show that there is dramatic heterogeneity in mutation dynamics across sites as a function of context.

The resampling test described in the Methods rejected the null hypothesis that there is uniformity in mutation dynamics across contexts. Comparing all pairs of matrices under the null hypothesis that the two matrices are drawn from the same underlying distribution leads to a rejection at the 5% level in 57.4% of the comparisons (Supplementary Fig. S1). This is even higher if the comparisons are limited to the matrix pairs in which each of them has a minimum of 50 substitutions. In this case the rejection at the 5% level is 71.8%. Not surprisingly, there is a higher rejection rate when matrices with dissimilar contexts, such as AA_AA and CC_CC (data not shown), are compared. Therefore, the variation observed in Fig. 1 is the result of a statistically significant difference in mutation dynamics across contexts.

### Observed and Expected Base Composition and Codon Usage

This wide heterogeneity in mutation dynamics across contexts has significant implications for the evolution of chloroplast genes. Most importantly for this study it means that there is no single expected base composition at all neutral sites in a gene. Rather, as noted above in the proline example, the expected base composition of a third codon position is a function of the first two codon positions of that same codon and of the neighboring 3′ codon. At the level of a gene, the composite composition of synonymous sites, and thus the CUB, is dependent on the amino acid sequence. As a result, even if it is assumed that compositional equilibrium, predicting expected codon usage in the absence of selection is far more problematic than often assumed. This is examined further here by comparing expected codon usage with and without a model incorporating context dependency of mutations.

**Fig. 2:**
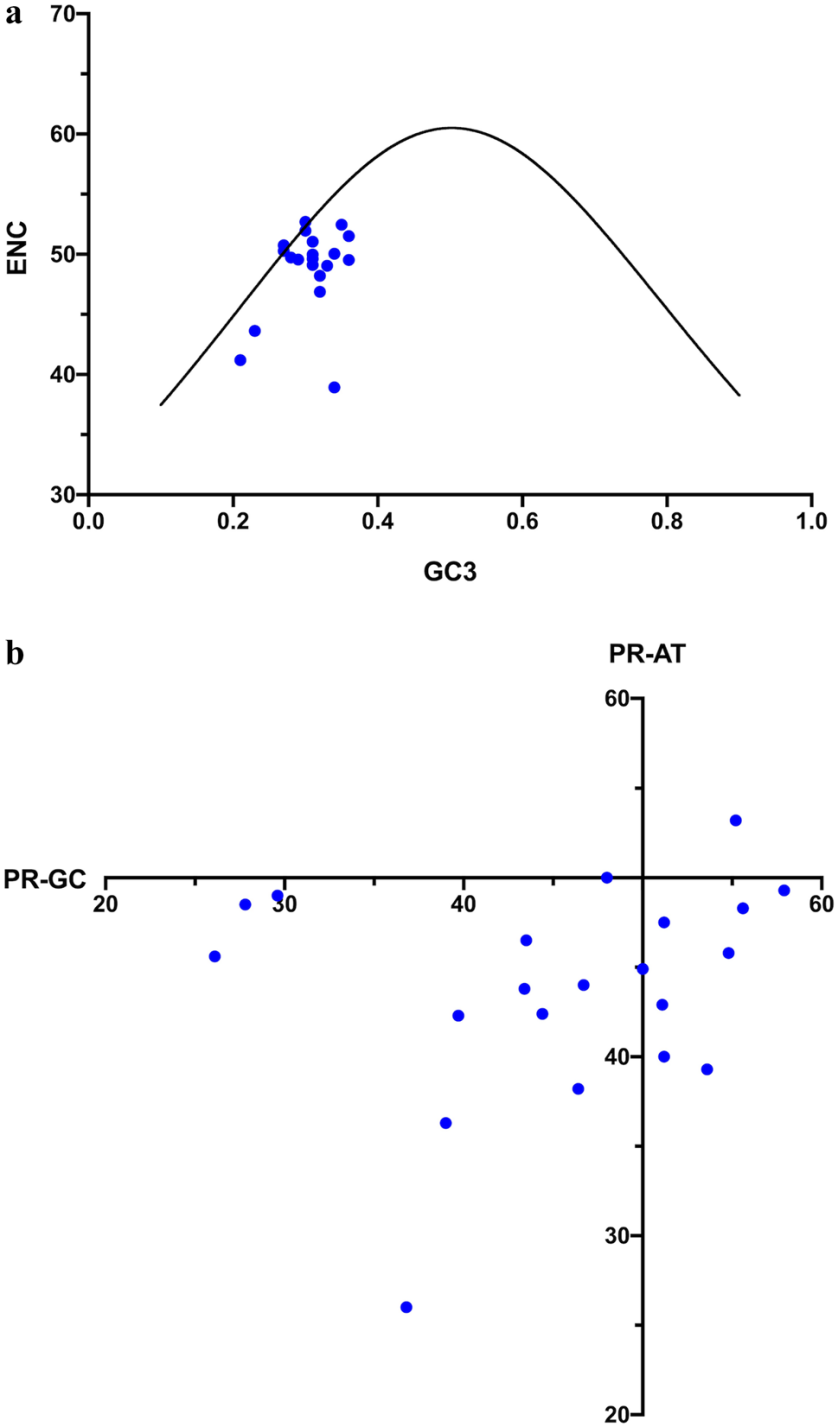
**a** Plot of ENC against GC3 for *Zea mays* chloroplast genes (blue) and ENC_E_ (black) following the formula from (Wright 1990). **b** Plot of PR-AT [N_A_/(N_A_ + N_T_)] against PR-GC [N_G_/(N_G_ + N_C_)] for *Zea mays* chloroplast genes. PR_E_ is the intersection of the axes where PR = 50; i.e., N_A_ = N_T_ and N_G_ = N_C (Color figure online)_

For the 21 *Zea mays*, chloroplast genes over 300 codons in length (*matK*, *psbB*, *psbC*, *rpoA*, *rpoB*, *rpoC1*, *rpoC2*, *atpA*, *atpB*, *psaA*, *psaB*, *petA*, *ccsA*, *ndhB*, *ndhD*, *ndhF*, and *ndhH*), ENC, PR-GC, and PR-AT values, the latter two at fourfold degenerate sites, are compared to ENC_E_ and PR_E_ (which is 50 for each of PR-AT and PR-GC) in Fig. 2. Not surprisingly, these genes deviate from the expectations in a pattern that is similar to what has been observed elsewhere for angiosperm chloroplast genes (Zhang et al. 2007, 2018; Xu et al. 2011; GuangXin et al. 2020; Liu et al. 2020; Duan et al. 2021).

**Fig. 3:**
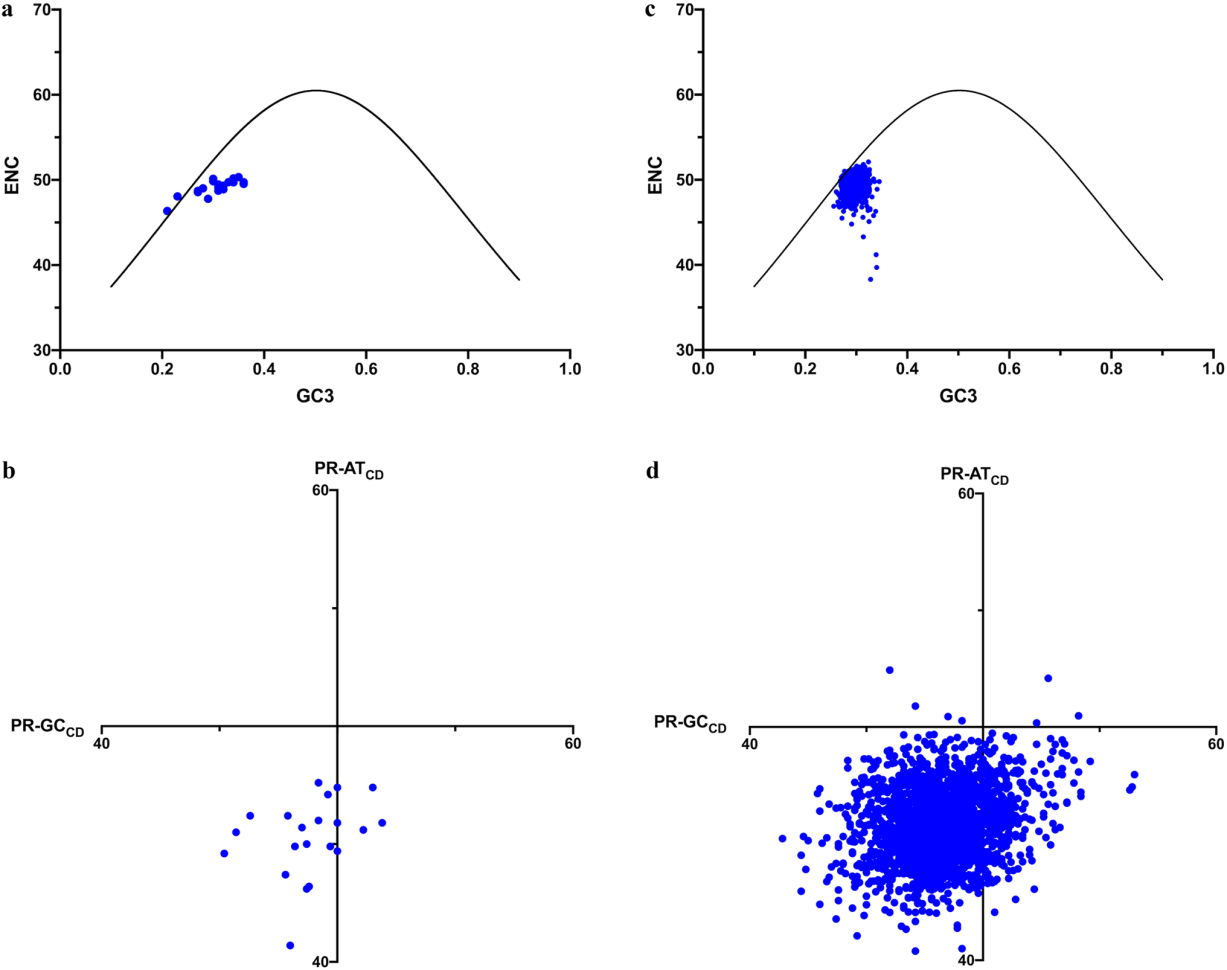
**a** ENC_CD_ for *Zea mays* chloroplast genes plotted against expected GC3 (blue) and ENC_E_ (black) following the formula from (Wright 1990). **b** ENC_CD_ for the 2000 *Zea mays* nuclear genes against expected GC3 (blue) and ENC_E_ (black) following the formula from (Wright 1990). **c** Plot of PR-AT_CD_ against PR-GC_CD_ for *Zea mays* chloroplast genes. **d** Plot of PR-AT_CD_ against PR-GC_CD_ for the 2000 *Zea mays* nuclear genes (Color figure online)

Given the heterogeneity of mutation dynamics, this work examined whether or not this deviation could be explained by the context-dependent mutation dynamics, as opposed to selection. To begin, the expected codon usage of each gene was calculated using the stationary vectors of the substitution matrices in combination with the amino acid sequence of the gene (see “Methods”). The ENC of this expected codon usage, which is the context-dependent expected ENC and will be called ENC_CD_, was calculated, as was the context-dependent expected Parity Rule values for fourfold degenerate sites, designated here as PR-AT_CD_ and PR-GC_CD_, or PR_CD_ generally. Unlike ENC_E_ and PR_E_, these values will be unique for each gene since they are a function of amino acid sequence. Figure 3A shows the ENC_CD_ values relative to ENC_E_ and Fig. 3B the PR_CD_ point for each gene.

To examine the properties of ENC_CD_ and PR_CD_ on a larger scale, 2000 genes were taken at random from the *Zea mays* nuclear genome, RefSeq ZmGDB181 at www.plantgdb.org, all greater than 300 codons in length to provide a large sample of actual amino acid sequences. For each, the ENC_CD_ and PR_CD_ as well as the expected GC3, was calculated all using the chloroplast mutation matrices as was described above for the chloroplast genes (Fig. 3C). The ENC_CD_ values show consistent deviation from the ENC_E_ curve. Genes fall primarily below the curve meaning that they will have a stronger bias in codon usage (lower ENC) than the G + C value predicts using the ENC_E_ equation (Wright 1990). Note that these data are not meant to predict CUB of nuclear genes, since mutation dynamics will differ in this genome, only to determine large scale trends in ENC_CD_ arising from chloroplast mutation dynamics in the absence of selection. Similarly, there is a consistent deviation from PR_E_ across genes in both PR-AT_CD_ and PR-GC_CD_ (Fig. 3D).

Overall, the data demonstrate that the use of ENC_E_ and/or PR_E_ is not valid as estimates of CUB in angiosperm chloroplast genes under neutrality. More appropriate are the codon usage predicted by ENC_CD_ and PR_CD_ which estimate neutral codon usage under the observed context-dependent mutation patterns.

### Comparing Genes to Expected Codon Usage and Base Composition

**Fig. 4:**
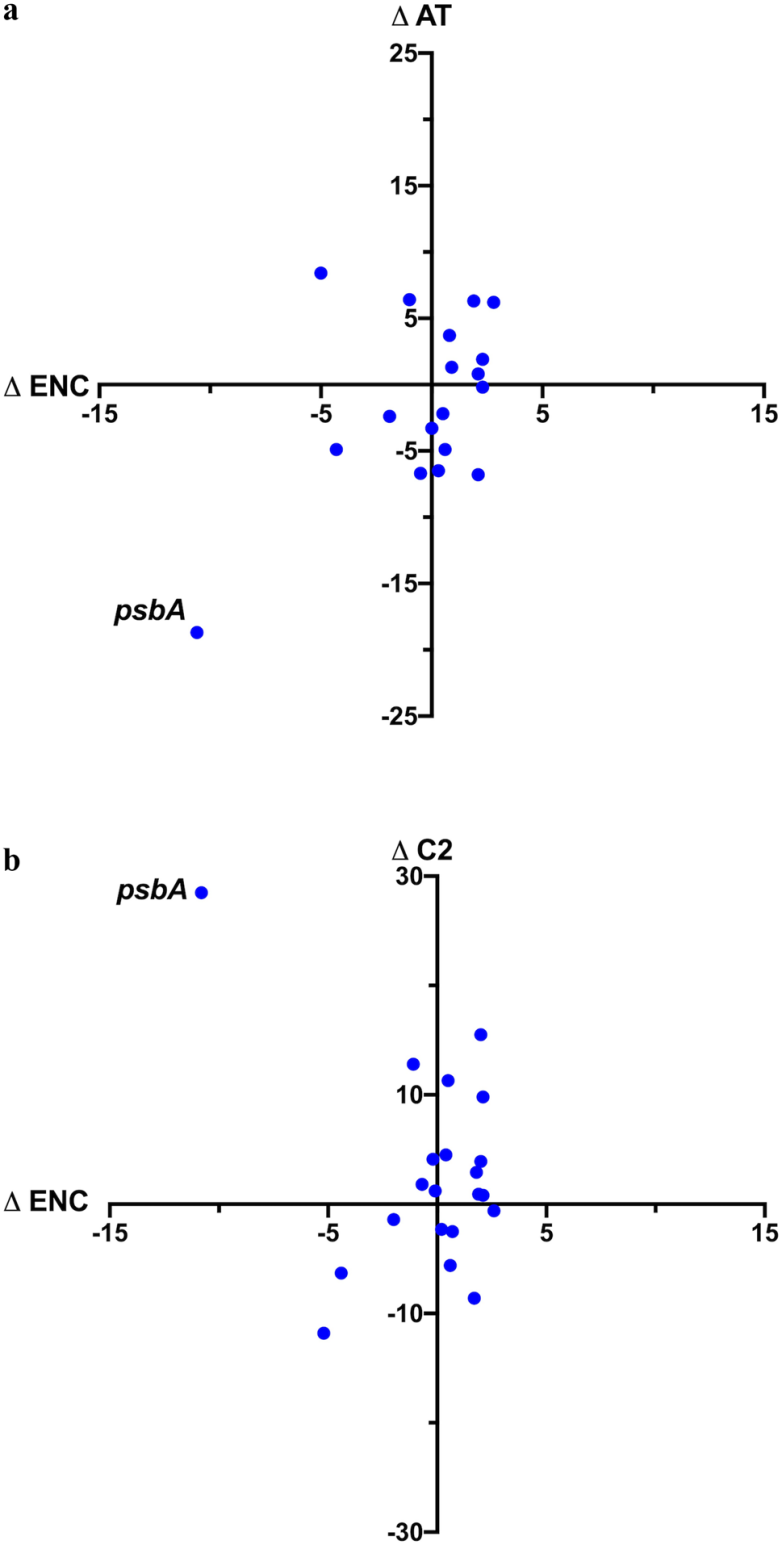
Difference between observed and expected codon usage in *Zea mays* chloroplast genes. The two composition features plotted are associated with adaptive codon usage as described in the text. **a** Difference in PR-AT and ENC. **b** Difference in C2 (%C at twofold degenerate NNY sites) and ENC (Color figure online)

The deviation of chloroplast genes from ENC_CD_, PR-AT_CD_, and PR-GC_CD_ is shown in Fig. 4. The deviation from expected percentage of C at twofold degenerate sites (C2_CD_) was also calculated since this is a feature of the presumptive adaptive CUB pattern observed in algae (Morton 1998; Suzuki and Morton 2016). The *psbA* gene shows substantially more deviation from the context-dependent expectations than other genes, and at an absolute level it is the only gene for which NNC is greater than 50% of the NNY codons. Additionally, *psbA* has a much stronger skew towards T at fourfold degenerate sites than any other gene.

To test for significance of the deviations observed in Fig. 4, the nested sampling described in the “Methods” was used, to account for sampling error in both the substitution matrices and the finite sequence length. For every chloroplast gene, over 300 codons in length from both the monocot *Zea mays* and the dicot *Arabidopsis thaliana* (the 21 genes listed above for *Zea* as well as *ycf1*, *ycf2*, and *accD*) 10^4^ gene sequences were generated, each using one of 100 sets of randomly resampled mutation matrices. From these sequences, the expected distributions of PR-AT_CD_, C_2CD_, ENC_CD_, and also calculated CAI_CD_ were generated in order to assess the directionality of codon usage bias. The CAI values were calculated using codon fitness values from highly expressed *Chlamydomonas reinhardtii* chloroplast genes and will measure the degree to which a gene uses codons that are optimal for translation efficiency in plastids (Suzuki and Morton 2016). Since cpDNA is strongly biased towards A + T the PR_CD_ analysis was limited to PR-AT_CD_.

**Fig. 5:**
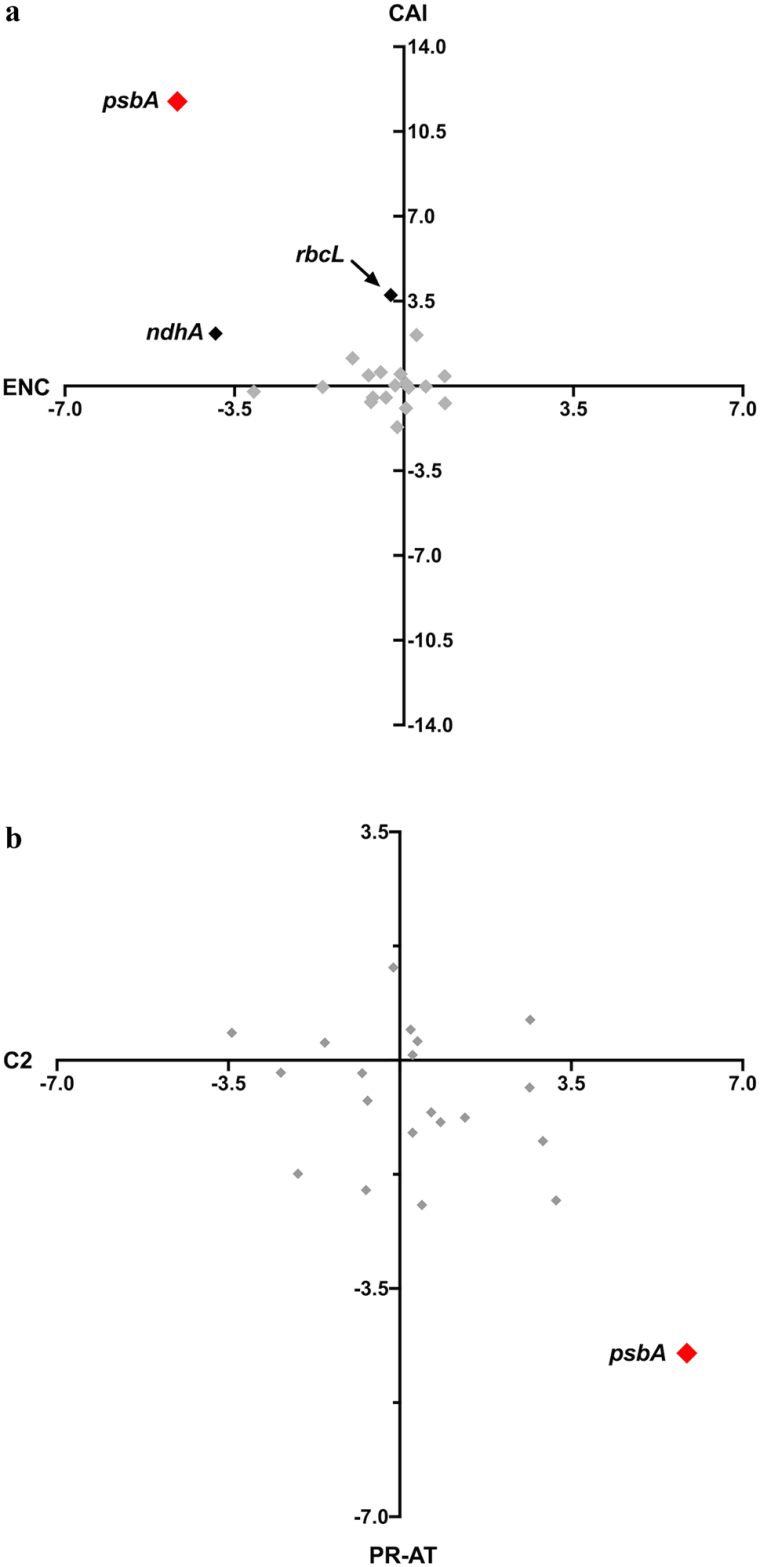
Number of standard deviations, the observed value is from the mean of the distribution from 10^4^ sequences generated by resampling for **a** ENC and CAI, **b** C2 and PR-AT. Genes that are significant (greater than 3.5 SD from the mean) along both axes are in red, genes significant along one axis are in black, and genes within the expected range are plotted in gray (Color figure online)

The results of this test are shown for the A + T matrices (see “Methods”) in Fig. 5 for the *Zea mays* genes. Significance at the 5% level with Bonferroni correction (Sokal and Rohlf 2009) is 3.5 standard deviations from the mean of the sampled gene sequences. The *psbA* gene shows significant deviation in both CAI and ENC meaning that it has a higher bias, specifically towards the adaptive CUB pattern, than predicted from the mutation dynamics. The *rbcL* gene is significant for CAI but not for ENC and no other gene shows significant deviation for either measure. The same general result is observed in *Arabidopsis* although the *psbD* gene also had a significantly higher CAI than expected. It is also apparent in Fig. 5 that both the PR-AT and C2 composition features are significant for *psbA*, not surprising since these two general features are associated with codon adaptation in *C. reinhardtii*, while all other genes have codon usage and composition features within the expected ranges as estimated from the resampling procedure. Repeating the analysis on *Zea mays* using the RY generic matrices gave the same general results as the A + T matrices (data not shown).

**Fig. 6:**
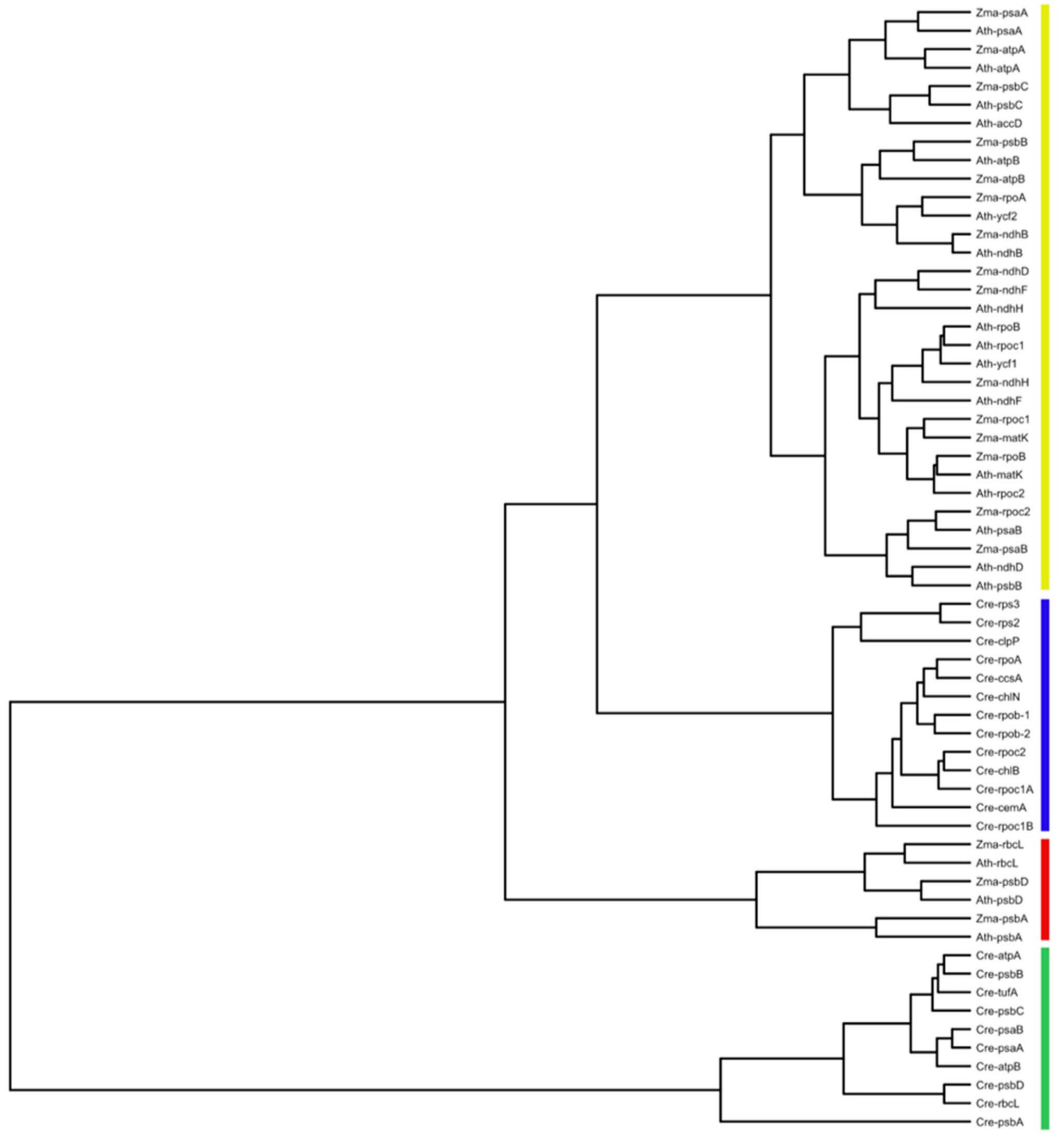
Cluster by similarity in codon usage of chloroplast genes greater than 300 codons in length from *Chlamydomonas reinhardtii* (Cre), *Zea mays* (Zma), and *Arabidopsis thaliana* (Ath). The four main clusters indicated are; highly expressed *Chlamydomonas* genes including *psbA* and *rbcL* (green), angiosperm *psbA*, *rbcL*, and *psbD* genes (red), other *Chlamydomonas* genes (blue) and other angiosperm genes (yellow) (Color figure online)

A cluster analysis was run on genes greater than 300 codons in length from the *Zea*, *Arabidopsis*, and *Chlamydomonas* chloroplast genomes (Fig. 6). This result highlights the difference between the codon usage of *psbA* and most other angiosperm chloroplast genes. Highly expressed *Chlamydomonas* genes form a distinct cluster, and the *psbA*, *rbcL*, and *psbD* genes from the two angiosperms form a separate cluster that is distinct from the other chloroplast genes. These clusters most likely arise from the different general CUB patterns described previously and quantified in Figs. 4 and 5; a bias towards C at twofold degenerate synonymous positions, and towards T at fourfold degenerate sites (Morton 1998, 2003).

## Discussion

Two general proposals have been put forward concerning the role of selection on codon usage in the angiosperm chloroplast genome. The first is that selection has a widespread influence, an assertion made based on observations that ENC values deviate from ENC_E_ and patterns of composition bias at fourfold degenerate coding sites deviate from PR_E_ (Zhang et al. 2007, 2018; Xu et al. 2011; GuangXin et al. 2020; Liu et al. 2020; Duan et al. 2021). The second proposal is that CUB is determined almost completely by mutation bias and genetic drift, with selection affecting primarily just the *psbA* gene to increase translation efficiency of this predominant translation product. This proposal has been based on the observation that *psbA* has an atypical codon usage pattern for angiosperm chloroplast genes, a pattern that matches the available tRNA population, that runs counter to the general composition bias, and that is similar to what is observed in highly expressed chloroplast genes in green algae (Morton 1998, 2003; Suzuki and Morton 2016).

These two models were tested taking into consideration the complex mutation dynamics of cpDNA. Inferring a role for selection based on deviation from ENC_E_ and the presence of compositional skew that fourfold degenerate sites assumes that these features do not arise from the underlying mutational process. The data presented here show that this assumption is false in the case of the angiosperm chloroplast genome. Instead, substitutions within intergenic regions show a very strong association with context, here defined as the base composition of the 4 nucleotides surrounding the mutation site. When the equilibrium base composition that would evolve within each context is calculated, a strong and statistically significant heterogeneity is observed. Expected G + C content, PR-AT, and PR-GC all range about four- to fivefold among contexts, and the variation is correlated with composition features of the context.

This compositional heterogeneity introduces complexities into predictions of codon bias in the absence of selection. Since each third codon position of a gene occurs within a specific context, which is determined by the amino acid sequence, different neutral sites will be evolving to very different compositional biases. Given the dramatic range in mutation dynamics across contexts it is clear that this is not something that can be ignored in molecular evolution. Models that assume identity across sites cannot be used to make predictions about expectations under neutrality.

The expected codon usage of each chloroplast gene was calculated based on the amino acid sequence of the gene and the observed context-dependent mutation dynamics and found that this predicted bias (ENC_CD_) showed noticeable deviation from ENC_E_ (Fig. 3a). Thus, ENC_E_, which is commonly taken to represent the expected CUB under neutrality, is not a valid statistic for angiosperm chloroplast genes. Rather, the ‘cloud’ of ENC_CD_ values in Fig. 3a represents the expected neutral CUB space; there is no single ENC value associated with one G + C content and each gene has a unique expectation. Therefore, deviation from ENC_E_ (Fig. 2a) cannot be taken alone as evidence for selection on codon usage. The complex mutation dynamics raise the same problem for the use of PR_E_; in the absence of selection, the mutation dynamics result in skew at fourfold degenerate sites (Fig. 3b).

Since ENC_E_ and PR_E_ are not accurate predictions of codon usage under neutrality, a nested resampling approach was used to test the deviation between observed codon usage and both ENC_CD_ and CAI_CD_, which are the expectations derived from the observed mutation dynamics. The resampling approach accounts for sampling error in the matrices as well as the finite sequence length. In the same test, PR_CD_ was compared to the observed skew at fourfold degenerate sites, as well as the observed and expected composition at twofold degenerate sites. The results show that codon usage and base composition features are within the predicted range for most chloroplast genes. The clear exception is *psbA* which deviates significantly from expectation (Fig. 5) in the compositional features associated with plastid codon adaptation and, consequently, in CAI itself. The other gene that showed a significant deviation was *rbcL* and, in the case of *Arabidopsis*, the *psbD* gene also showed a significantly higher CAI than expected. The distinct nature of codon usage of *psbA* and, to a lesser extent, *rbcL* and *psbD* are also apparent in the cluster analysis (Fig. 6). Four distinct gene clusters can be seen, one consisting of set of genes that includes the highly expressed *psbA* and *rbcL* genes, from *Chlamydomonas*, another the *psbA*, *rbcL*, and *psbD* genes from *Zea* and *Arabidopsis*, with the rest of the chloroplast genes forming a large cluster within which the remaining *Chlamydomonas* genes form a distinct group, possibly due to a difference in mutational dynamics between algae and flowering plants.

The results overall strongly support the model that CUB of angiosperm chloroplast genes is largely determined by mutation bias with selection playing a limited role, affecting only the *psbA* gene to a noticeable degree. The evidence here also suggests that the *rbcL* and *psbD* genes might be under very weak selection for translation efficiency. The findings are consistent with the observation from transcriptome data that *psbA* and *rbcL* are the major expression products, along with the ribosomal RNAs, of the chloroplast (Castandet et al. 2016).

One complexity that context dependency raises, and that was not taken into account here, is that the context of a third codon position will change over time as the amino acid sequence of a gene evolves. This is a very difficult problem to address so it has been assumed that the third codon positions are close to equilibrium. The finding that expected and observed match quite well suggests that this factor is not confounding the analysis. However, a future comparison of conserved and variable amino acid sites across the chloroplast genome and angiosperm evolution might help shed some light on the possible influence of nonequilibrium on molecular studies.

The data show that it is unnecessary to invoke selection to explain the codon usage of most angiosperm chloroplast genes. However, one possibility that must be considered is that there is genome-wide selection on base substitutions that is unrelated to codon usage. Such selection would influence the derived substitution matrices but the matrices would still accurately predict codon usage patterns. This scenario seems highly unlikely. It would require that selection influences essentially every substitution in the chloroplast genome. Moreover, when matrices from intergenic regions are compared to those from fourfold degenerate coding sites strongly correlated patterns are observed (data not shown) similar to what has been found previously (Morton 1997, 2003). Therefore, whatever the putative selective pressure is, it would need to affect every site in the genome in the same way, coding or noncoding. It is unclear what type of selection would act this way and generate the observed context-dependent dynamics. The most likely explanation is simply that the observed substitution pattern is an accurate model of the underlying mutation process in angiosperm cpDNA.

Overall, the results show that caution must be used when drawing conclusions about selection on angiosperm chloroplast genes. The use of composition data without a consideration of the real and complex mutation dynamics will be misleading. When context dependency is accounted for the evidence indicates that selection plays a limited role in determining CUB of these genes. The data also indicate that the use of composition data should be approached with caution in every gene and a rigorous analysis of mutation dynamics be undertaken.

## Supplementary Information

Below is the link to the electronic supplementary material.

Supplementary file1 (PDF 63 KB) Results of the resampling test for substitution matrix homogeneity. For each pair of contexts (32,640 pairs in total) the two matrices were tested for the null hypothesis that they are drawn from the same set of substitutions. An expected matrix distance distribution was generated from iterations of resampling with replacement from the pooled matrix to generate two new matrix pairs. The *P* value is 1 minus the proportion of resampled pairs with a lesser distance than the original. A *P* value of 0 indicates that all resampled matrix pairs were more similar than the original pair. The frequencies of binned *P* values from the 32,640 tests are shown here. Each bin represents the tests with a *P* value less than the axis value but greater than the axis value to the left. The frequency below 0.05, the left two bins, was 57.3%.

## Declarations

### Data Availability

All data will be made available upon reasonable request.

### Code Availability

All custom code will be made available upon reasonable request.
