# Supplementary material for: Context-Dependent Mutation Dynamics, Not Selection, Explains the Codon Usage Bias of Most Angiosperm Chloroplast Genes
Source: J Mol Evol. 2021 Dec 21;90(1):17–29. doi: 10.1007/s00239-021-10038-w (PMC8821512; doi:10.1007/s00239-021-10038-w)
Supplement: Supplementary file 1 — Supplementary file1 (PDF 63 KB) Results of the resampling test for substitution matrix homogeneity. For each pair of contexts (32,640 pairs in total) the two matrices were tested for the null hypothesis that they are drawn from the same set of substitutions. An expected matrix distance distribution was generated from iterations of resampling with replacement from the pooled matrix to generate two new matrix pairs. The P value is 1 minus the proportion of resampled pairs with a lesser distance than the original. A P value of 0 indicates that all resampled matrix pairs were more similar than the original pair. The frequencies of binned P values from the 32,640 tests are shown here. Each bin represents the tests with a P value less than the axis value but greater than the axis value to the left. The frequency below 0.05, the left two bins, was 57.3%. [file 239_2021_10038_MOESM1_ESM.pdf]

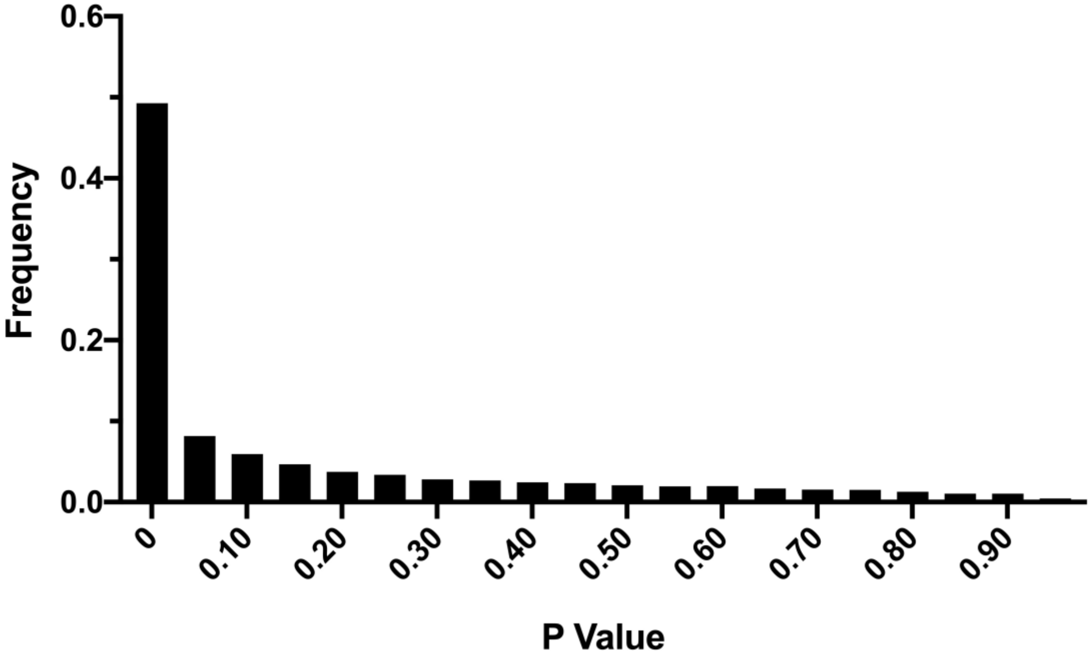

Figure S1. Results of the resampling test for substitution matrix homogeneity. For each pair of contexts (32,640 pairs in total) the two matrices were tested for the null hypothesis that they are drawn from the same set of substitutions. An expected matrix distance distribution was generated from iterations of resampling with replacement from the pooled matrix to generate two new matrix pairs. The P value is 1 minus the proportion of resampled pairs with a lesser distance than the original. A P value of 0 indicates that all resampled matrix pairs were more similar than the original pair. The frequencies of binned P values from the 32,640 tests are shown here. Each bin represents the tests with a P value less than the axis value but greater than the axis value to the left. The frequency below 0.05, the left two bins, was 57.3%.
